# Geopolymer Recycled Aggregate Concrete: From Experiments to Empirical Models

**DOI:** 10.3390/ma14051180

**Published:** 2021-03-03

**Authors:** Hoai-Bao Le, Quoc-Bao Bui, Luping Tang

**Affiliations:** 1 Sustainable Developments in Civil Engineering Research Group, Faculty of Civil Engineering, Ton Duc Thang University, Ho Chi Minh City 700000, Vietnam; lehoaibao.st@tdtu.edu.vn; 2 Civil Engineering Faculty, Mien Tay Construction University, Vinh Long 85100, Vietnam; 3 Department of Architecture and Civil Engineering, Division of Building Technology, Chalmers University of Technology, 41296 Gothenburg, Sweden; tang.luping@chalmers.se

**Keywords:** recycled aggregate concrete (RAC), geopolymer, fly ash, modified Feret’s model, De Larrard’s model

## Abstract

Ordinary cement concrete is a popular material with numerous advantages when compared to other construction materials; however, ordinary concrete is also criticized from the public point of view due to the CO_2_ emission (during the cement manufacture) and the consumption of natural resources (for the aggregates). In the context of sustainable development and circular economy, the recycling of materials and the use of alternative binders which have less environmental impacts than cement are challenges for the construction sector. This paper presents a study on non-conventional concrete using recycled aggregates and alkali-activated binder. The specimens were prepared from low calcium fly ash (FA, an industrial by-product), sodium silicate solution, sodium hydroxide solution, fine aggregate from river sand, and recycled coarse aggregate. First, influences of different factors were investigated: the ratio between alkaline activated solution (AAS) and FA, and the curing temperature and the lignosulfonate superplasticizer. The interfacial transition zone of geopolymer recycled aggregate concrete (GRAC) was evaluated by microscopic analyses. Then, two empirical models, which are the modified versions of Feret’s and De Larrard’s models, respectively, for cement concretes, were investigated for the prediction of GRAC compressive strength; the parameters of these models were identified. The results showed the positive behaviour of GRAC investigated and the relevancy of the models proposed.

## 1. Introduction

The consumption of concrete in the construction sector is increasing, which causes an increase in cement production and natural resource exploitation. The depletion of the natural resources and the CO_2_ emissions are problems which need to be treated for a sustainable development. To reduce the natural resource exploitation, recycling is a strategy which has been mentioned and is encouraged; on the other hand, the reduction in carbon footprint for concrete material is still a challenge. Indeed, the cement industry is one of the major sectors which generates CO_2_ [1]. The production of one ton of Portland cement emits approximately one ton of CO_2_ into the atmosphere; the cement manufacturing contributes 7% to the global CO_2_ emission [2,3]. It is important to find alternative binders which have a lower carbon footprint than cement. One of the promising alternatives is to use industrial by-products (such as fly ash, slag, etc.) to replace partially or totally the cement in concrete. The total replacement of cement is also possible by using geopolymer as a binder [4]. Davidovits [4] proposed the use of alkaline activators (composed of OH ions such as NaOH, KOH, or Mg(OH)_2_, and SiO_2_ ions such as Na_2_SiO_3_) to react with materials containing Si and Al ions (such as fly ash, slag) to produce geopolymer. The term “geopolymer” is used for the binder obtained because the occurred chemical reactions are a polymerization process. The application of geopolymer in the production of concrete (called “geopolymer concrete”) has been investigate in numerous previous studies by replacing cement by geopolymer [5].

In recent decades, important amounts of construction and demolition (C&D) wastes have been produced due to urbanization. The wastes from demolished concretes cause environmental issues when disposed in landfill sites [6]; however, these demolished concrete wastes can be recycled to be used as construction materials [7]. The recycling of C&D wastes has positive effects on the environmental and the economic aspects, so numerous investigations have been carried out on this topic [6,7,8,9,10,11]. The old concrete is crushed, sieved, and cleaned to become recycled concrete aggregates. These recycled aggregates can be used as substitutes (partially or completely) of natural aggregates (coarse or/and fine) [7]. The recycled aggregates usually contain natural aggregates bonded with old cement mortars. The old mortar increases the porosity of the recycled aggregate concrete (RAC) compared to natural aggregate which decreases the mechanical characteristics of RAC obtained. In RAC, there are two types of interfacial transition zones (ITZ): between the old mortar and the parent natural aggregates, and between the recycled aggregates and the new mortar. The ITZ between the old mortar and the new mortar contributes also to the decrease in mechanical characteristics of RAC; the improvement of this type of ITZ is an interesting topic to be explored [7].

While the number of studies on geopolymer concrete (with natural aggregates) or RAC (with Portland cement) is high, the number of investigations on geopolymer recycled aggregate concrete (GRAC) is still limited, although some studies have been initiated [12,13]. These previous studies in the literature on GRAC have evaluated different aspects on the mechanical properties of GRAC; however, the effects of curing temperatures, superplasticizers, and the models for the strength prediction have not yet been reported. It is worth mentioning that there have already been numerous studies on effects of the curing temperature and superplasticizers on geopolymer concrete with natural aggregates, but these effects in the case of recycled aggregates have not yet been reported in the literature to our knowledge. In the present study, first, a parametric study was performed to propose the optimized mixes for the GRAC containing 100% recycled coarse aggregates; influences of the curing temperature and the superplasticizer have been investigated. Then, two empirical formulas (modified version of Feret’s model and De Larrard’s model for ordinary cement concretes) were applied and the identification of the empirical parameters for the models was conducted. The models validated can provide useful information for the compressive strength prediction of GRAC and the corresponding mix design.

## 2. Experiments

### 2.1. Materials Used

#### 2.1.1. Fly Ash

**Figure 1:**
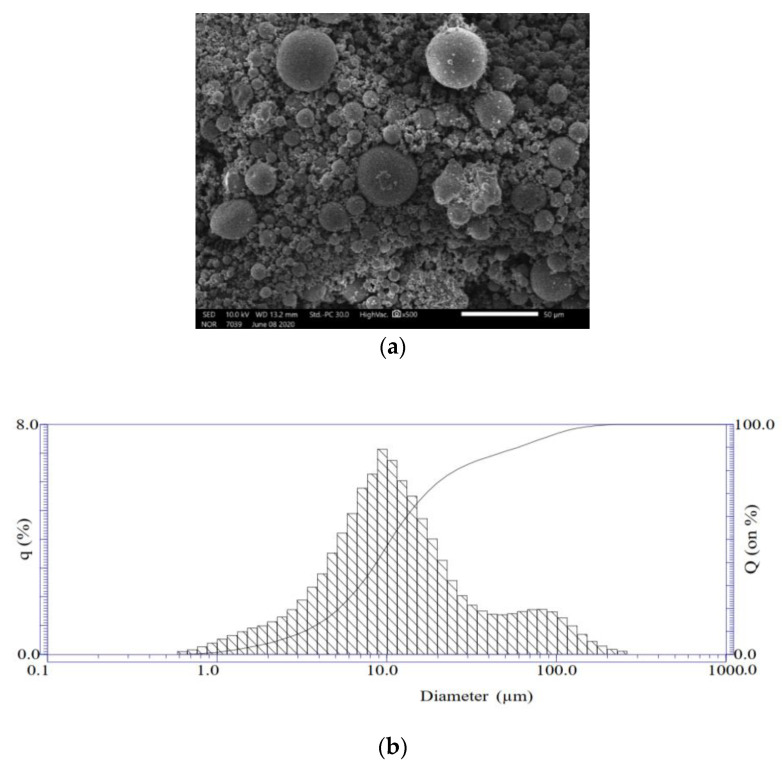
(**a**) SEM of fly ash (FA) particles; (**b**) Size of FA particles varying from 0.6 to 250 µm.

Fly ash (FA) used in the present study was collected from the DH3 Thermal Power Plant, located in southern Vietnam. The analyses of the particle size distribution by scanning electron microscope (SEM, JSM-IT200, Jeol, Tokyo, Japan) showed that the FA used has spherical form (Figure 1a) with dimensions varying from 0.6 to 250 µm; the mean dimension is about 10 µm (Figure 1b).

The chemical composition of the FA was determined following ASTM 618 procedure [14]. The result is presented in Table 1, which shows that the used FA corresponds to Class-F following ASTM classification. The contents of aluminum oxide (Al_2_O_3_) and silicon oxide (SiO_2_) are high enough for the alkali-activation of the binder. The specific density of FA was also measured; a value of 2.44 was obtained.

#### 2.1.2. Aggregates

**Figure 2:**
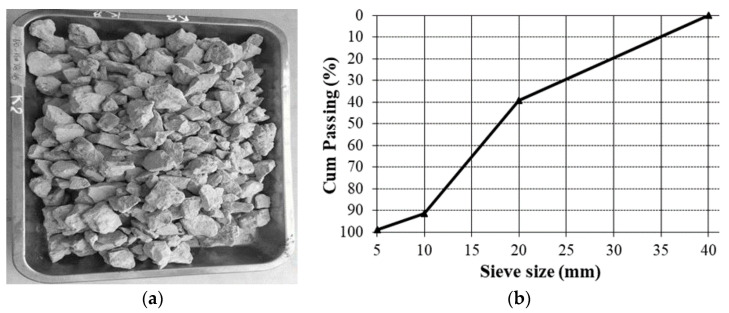
(**a**) Recycled coarse aggregate used and (**b**) the particle size distribution.

Several previous studies suggested limiting the substitution of natural sand by recycled fine aggregate; the maximum substitution ratio of 30% is currently proposed, due to the durability concerns of recycled aggregate concretes [7]. In the present study, only coarse aggregate was substituted by recycled coarse aggregate. The coarse aggregate used in this study was 100% recycled from an old ordinary concrete. It was shown in several studies that the quality of the parent concrete did not directly influence the mechanical characteristics of the recycled aggregate concrete obtained; the important parameter was the quality of the recycled aggregates obtained [7,15]. The recycled aggregate was obtained by crushing the parent concrete and taking the portion sieved from 5 to 30 mm, which were the current dimensions of coarse aggregate for ordinary concretes in Vietnam. Figure 2 illustrates the appearance and size distribution of recycled coarse aggregate obtained.

**Figure 3:**
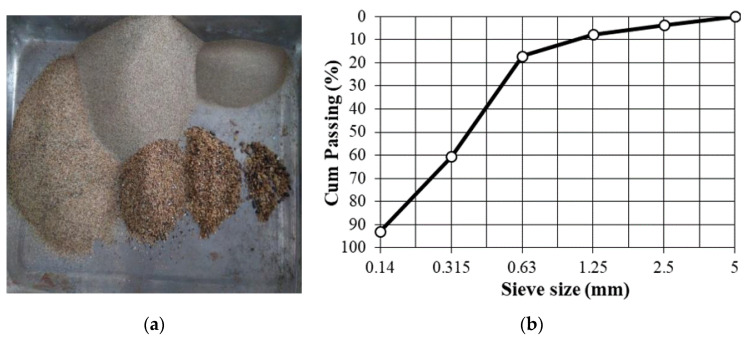
(**a**) Natural fine aggregate used and (**b**) its particle size distribution.

The fine aggregate used in the present study was a natural river sand. The sand was of class 0/5 (nominal maximum size of 5 mm). The particle size distribution tests were carried out and the result is illustrated in Figure 3. The fineness modulus of the sand used was of 1.8, which corresponded to a fine sand for concrete manufacturing.

#### 2.1.3. Characterization of Recycled Coarse Aggregate (RCA)

Different parametric tests have been performed to determine the specific gravity, the dry density, the saturated density, and the water absorption following the Vietnamese standard [16]. The results are presented in Table 2. The corresponding characteristics of a natural coarse aggregate which is currently used for ordinary concrete in the region are also presented in Table 2 for a comparison. The specific gravity (or relative density) is defined as the ratio of the mass of a unit volume of the aggregate (without void) to that of water. Other densities are also presented as ratio to the density of water.

The specific gravity of the natural coarse aggregate was of 2.66; the specific gravity of the RCA obtained was of 2.60. There was a slight decrease in the specific gravity of recycled aggregates when compared to the natural aggregate. The presence of old mortar attached to the RCA is responsible for the lower value of specific gravity of RCA compared to natural coarse aggregate. Furthermore, the microstructure of the recycled aggregates may be degraded during the recycling (such as the crushing). This result had also been observed in several previous studies [17,18]. The same observation is noted for the dry density because the recycled aggregates are usually bonded with old paste which are more porous than natural aggregate. It is well-known that the recycled aggregates have higher porosity than the corresponding natural aggregate, which causes a lower dry density and a higher water absorption than natural aggregates [19].

#### 2.1.4. Aggregate Crushing Value (ACV)

**Figure 4:**
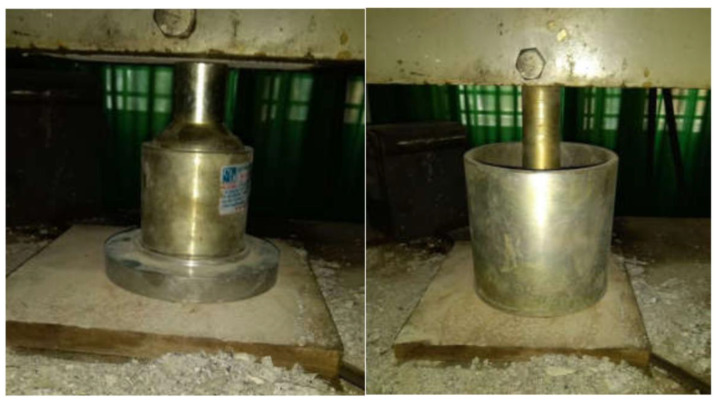
Test for aggregate crushing value.

The strength of bulk coarse recycled aggregate was evaluated by determining the ACV following the Vietnamese standard [20]. A steel cylinder (15 cm-diameter) was filled with aggregates having specified size distribution and volume (Figure 4). The weight of the aggregate sample was M_1_. A plunger was inserted into the cylinder and a load was applied at a uniform rate of 40 kN/min up to 400 kN. After removing the load, the aggregate was removed and sieved over a 2.36 mm sieve; the passing aggregate particles were weighed (called M_2_). The aggregate crushing value was calculated by:

ACV = M_2_/M_1_, (1)

The ACV can be determined for aggregates at dry state or saturated state. In the present study, ACV was determined for saturated state. The results obtained are presented in Table 2.

The ACV results of this study are a similar range to that in the literature [21,22]: the natural aggregate has lower ACV (corresponding to a higher strength) than recycled aggregates. The compressive strength of aggregates can be deduced from the ACV by empirical relationships [20]; in this case, the corresponding compressive strength deduced were of 70 and 34 MPa, respectively, for natural aggregate and recycled coarse aggregate. This result shows a significant decrease in compressive strength of recycled aggregates. It is worth noting that the mechanical properties obtained of the recycled aggregates used in the present study are similar to several other recycled aggregates used in previous studies in the literature [21].

#### 2.1.5. Alkali-Activated Binder

To activate the fly ash, a combination of sodium hydroxide solution (NaOH) and sodium silicate solution (Na_2_SiO_3_) was chosen as the AAS which are also the current substances used in previous studies to produce geopolymer [23,24]. Hardjito and Rangan [25] indicate that higher molar concentration of NaOH solution provides higher compressive strength of geopolymer concrete, and the effect of Na_2_O/Si_2_O molar ratio in Na_2_SiO_3_ on the compressive strength of geopolymer concrete is negligible. A NaOH of 99% purity and a Na_2_SiO_3_ with 11.8% Na_2_O, 29.5% SiO_2_, and 58.7% water was used. The NaOH solution was prepared by dissolving either the flakes or the pellets in water. The mass of NaOH solids in the solution varied depending on the concentration of the solution expressed in terms of molar, M. In the present study, NaOH solution with a concentration of 12 M contains 12 × 40 = 480 g of NaOH solids (in flake or pellet form) per liter of the solution, where 40 is the molecular weight of NaOH [25]. The mass of NaOH solids was measured as 361 grams per kg of NaOH solution of 12 M concentrations. Na_2_SiO_3_ and NaOH solutions were prepared one day prior to usage.

#### 2.1.6. Superplasticizer

The influences of superplasticizers on the workability of FA geopolymer concrete were investigated in a previous study [25]. It was shown that not all superplasticizers could improve the performance of FA geopolymer concrete: with polycarboxylic-based superplasticizers, no significant difference in the performance of FA geopolymer concrete was observed; however, by adding 2% of naphthalene sulfonate-based superplasticizer (compared to FA), the workability was improved but the compressive strength was not affected. In the present study, a lignosulfonate superplasticizer (Sika, Baar, Switzerland) was used because lignosulfonate and naphthalene sulfonate are considered the most widely used superplasticizers [26]. The used superplasticizer was a current commercial product in liquid form. The amount of superplasticizer was 2% of the FA, similar to the previous study [25].

### 2.2. Mix Proportions for GRAC

In the literature, the Na_2_SiO_3_/NaOH ratio recommended for FA geopolymer concrete (with natural aggregates) was of 2.5 [25]. The present study followed also this recommendation for GRAC: the Na_2_SiO_3_/NaOH ratio was kept constant at 2.5, the NaOH concentration was of 12 M, the superplasticizer was equal to 2% of FA (by mass). It was observed in previous studies that when the AAS/FA ratio increased (where AAS was alkali-activator solution), the workability increased but the compressive strength decreased; the recommended values of AAS/FA for geopolymer concrete with natural aggregates ranged from 0.3 to 0.45 (by mass) [27]. Since recycled aggregates have higher water absorption than natural aggregates, to maintain the same workability as geopolymer concrete with natural aggregate, the AAS/FA ratio should be increased in the case of GRAC. Therefore, the present study tested AAS/FA ratios at 0.4, 0.45, and 0.5. The mix proportions of GRAC are shown in Table 3.

For further comparisons, specimens of control mixtures were also manufactured which contained natural coarse aggregates in place of RCA. The control mixtures did not contain superplasticizer and will be compared with GRAC without superplasticizer.

### 2.3. GRAC Specimen Casting

**Figure 5:**
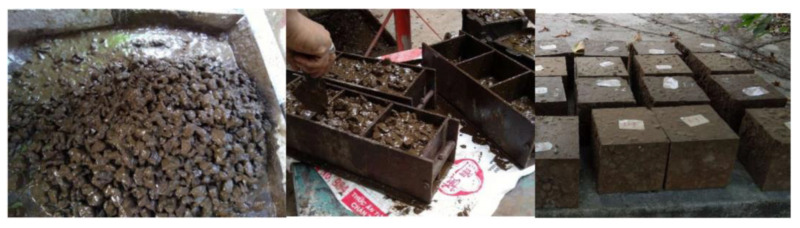
Manufacturing procedure of geopolymer recycled aggregate concrete (GRAC) specimens.

First, the fine aggregate and the recycled coarse aggregate (at initial state, which was a quasi-dry state) were mixed in a mixer (Matest, Italy) for 1–2 min followed by the addition of FA to the mixer and mixed for further 2–3 min. Alkali-activated solutions were then progressively poured into the mixer where they were mixed for further 2–3 min until a uniform mixing was observed. Superplasticizer were added at the same time as the alkali-activated solution. After the mixing, the concrete specimens were casted in 15 × 15 × 15 cm^3^ cube molds and compacted on a vibrating table. Figure 5 shows the manufacturing process of geopolymer concrete specimens.

### 2.4. Curing

Another parameter which was investigated in this study was the curing temperature. Studies reported in the literature showed that FA based geopolymer concrete could be cured under ambient conditions like ordinary cement concrete, but a higher curing temperature could significantly support the chemical reactions occurring in geopolymer, which provides a higher compressive strength [28]. However, the increase in curing temperature beyond 60 °C does not significantly increase the compressive strength [25] but just consumes more energy. That was why in the present study two types of curing were investigated: in the first one, the specimens were cured under laboratory ambient conditions (27 °C and 60% RH); in the second type, the specimens were cured at 60 °C for 24 h. For the curing with elevated temperature, the specimens were first cured in the mould under ambient conditions for 24 h (27 °C and 60% RH), then demolded and placed in an oven at 60 °C for 24 h; after the heating curing, the specimens were placed in the laboratory to cool down. All specimens were stored in a controlled room (27 °C and 60% RH) until the testing ages (3, 7, 14, and 28 days).

### 2.5. Geopolymer Mortar and Paste

**Figure 6:**
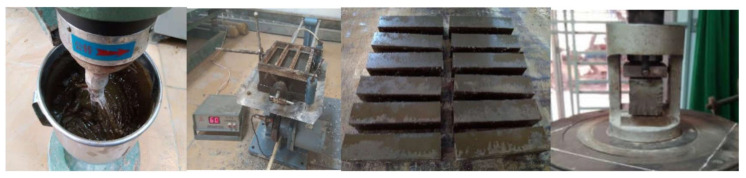
Confection and testing of geopolymer mortar/paste specimens.

The compressive strength of geopolymer paste is an important parameter for the compressive strength of the geopolymer concrete obtained. To provide this parameter in the model of compressive strength prediction, specimens with dimensions of 4 cm × 4 cm × 16 cm were manufactured (Figure 6). Since there have not yet been standard for the tests on geopolymer mortar/paste, three different compositions have been tested:

- Geopolymer paste which does not contain sand: only FA and AAS were used, by using the AAS/FA ratio of 0.4, which was the same for the case of GRAC.
- Geopolymer mortar which contains natural sand but does not contain any coarse aggregate: the proportion of FA, AAS, and sand was the same as the case of GRAC.
- The third type (called “mortar-standard”) is a geopolymer mortar which contains the sand but the proportion between binder (FA + AAS) and sand respected the proportion proposed for cement mortar following standard.

The summary of the proportions applied are illustrated in Table 4. For each test of each composition (at different ages), three specimens were manufactured and tested. The manufacturing procedure is summarized in Figure 6.

Similar to GRAC specimens, the mortar/paste specimens were also cured in two different conditions: at ambient conditions or heated at 60 °C during 24 h; the specimens were also tested at 3, 7, 14, and 28 days. For each mortar/paste specimen (4 cm × 4 cm × 16 cm), first, a 3-point-bending test was performed to obtain 2 specimens of 4 cm × 4 cm × 8 cm. Then, uniaxial compression test was performed to determine the compressive strength of these specimens (Figure 6).

## 3. Results

### 3.1. Workability of GRAC

**Figure 7:**
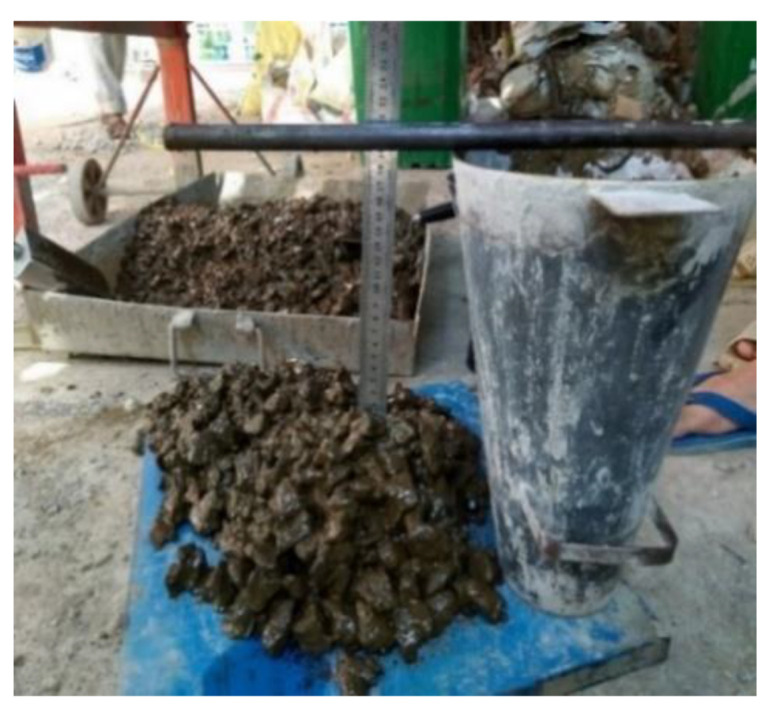
Slump measurement of fresh concrete.

**Figure 8:**
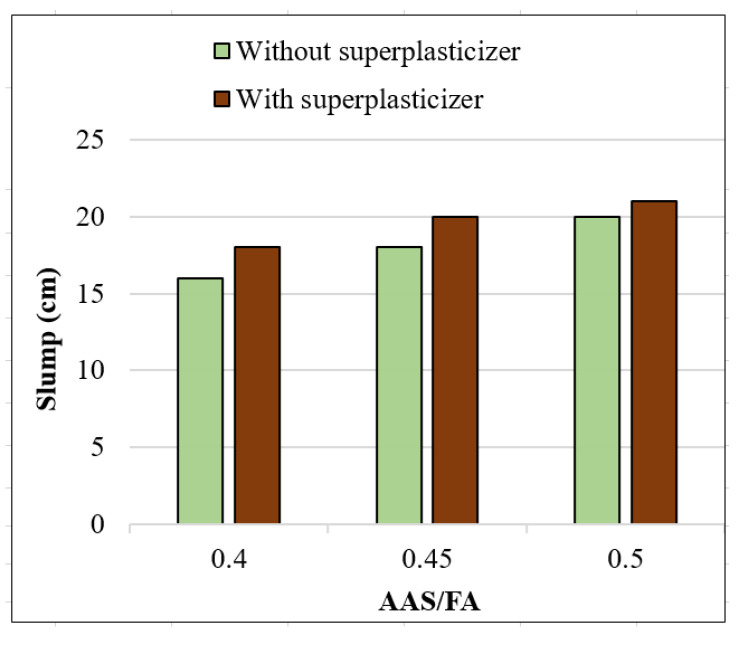
Slump values of GRAC in function of alkaline activated solution/fly ash AAS/FA ratios.

The workability of the fresh concrete was measured by the conventional slump test (Figure 7) [29]. The slump test is a simple and current method for the workability assessment of ordinary concrete; this method is also applied for geopolymer concrete [5,25]. The results are shown in Figure 8 which indicates that the slump values reduced when the AAS/FA ratio decreased. This result is not surprising because with a higher AAS/FA ratio, the quantity of AAS (form of liquid) increases, which increases the workability. For the case without lignosulfonate superplasticizer, the slump values were of 16, 18, and 20 cm for AAS/FA of 0.4, 0.45, and 0.5, respectively. When the lignosulfonate superplasticizer was used, the slumps increased to values from 18 to 21 cm. Thus, the lignosulfonate superplasticizer increased by about 2 cm of slump for each corresponding mix. For all mixes tested, the slumps respected the minimum value (18 cm) which is usually recommended for geopolymer concretes [30,31].

It is worth mentioning that due to the liquid-form of the binder (alkali-activated solution), in the fresh state the alkali-activated solution “slumps” more easily than the aggregates, which causes a certain separation between the aggregate and the binder (Figure 7). The effects of the superplasticizer used on the compressive strength and explanations about the role of superplasticizer are discussed in the next section.

### 3.2. Compressive Strength of GRAC

**Figure 9:**
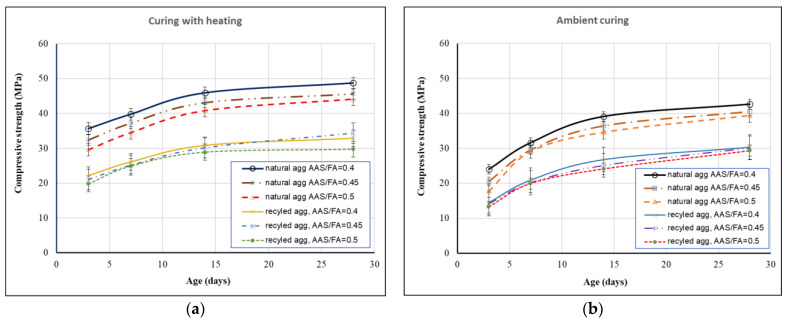
Compressive strength of GRAC and control mixture without superplasticizer; (**a**) cured at 60 °C; (**b**) cured at ambient conditions.

**Figure 10:**
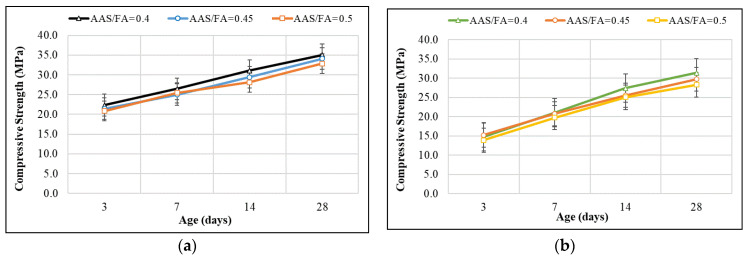
Compressive strength of GRAC with superplasticizer; (**a**) cured at 60 °C; (**b**) cured at ambient conditions.

The compressive strength of GRAC and control mixture (with natural coarse aggregates) were determined at the ages of 3, 7, 14, and 28 days in accordance with the current standard [32]. The results obtained were the mean values of three specimens tested. The results obtained from the uniaxial compression tests are illustrated in Figure 9 and Figure 10. From Figure 9, it is observed that when compared to the control mixture (geopolymer concrete with natural aggregate), GRAC had lower compressive strength (about 30%) for all AAS/FA ratios tested. This result is expected due to lower mechanical characteristics of recycled aggregates compared to natural aggregates as shown in the previous section. For both Figure 9 and Figure 10, the curves show a general evolution of the compressive strength in function of time. For specimens cured under ambient conditions (Figure 9a and Figure 10a), the compressive strength development at the early age (3 days) is similar to that of ordinary cement concretes: the 3-days compressive strength is about 50% of 28-days compressive strength [33]. For specimens cured under 60 °C for 24 h (Figure 9b and Figure 10b), a quicker increase in compressive strength at early age is observed: the compressive strength at 3 days is about 60% of that at 28 days. This result shows influence of the thermal curing on the early age strength development.

From Figure 9 and Figure 10, the difference of the results obtained with three AAS/FA ratios tested is clear for control mixtures (geopolymer natural aggregate concrete) while the difference is low for GRAC (less than 6% for all cases). Among the three AAS/FA ratios tested, the best ratio should be AAS/FA = 0.4 because this ratio provides almost the highest compressive strength, and this ratio is the most economical when the use of AAS is lowest. For the case of AAS/FA = 0.4, the compressive strength at 28 days obtained on ambient curing GRAC specimens was about 30–31 MPa (Figure 9a and Figure 10a), while it was about 34–35 MPa for GRAC specimens cured under 60 °C during 24 h (Figure 9b and Figure 10b). This result is equivalent to an increase of 10% of compressive strength thanks to 24 h-heating curing. These 28-days-mean compressive strengths are equivalent to C20 and C25 concretes following Eurocode 2 [34], which means that the GRAC investigated may satisfy the criteria about the compressive strength for practice applications. It is worth noting that the recycled coarse aggregate used in the present study had a compressive strength of 34 MPa (Section 2.1.3 above), so the compressive strength of the GRAC obtained would be limited to this value. It is suggested that if a better recycled aggregate was used, the compressive strength of the concrete obtained would be higher because of the higher strength of the geopolymer matrix; this point will be seen later in Section 3.4.

The effect of lignosulfonate superplasticizer on the compressive strength obtained is not significant. Similar results were observed in previous studies with other types of aggregate using geopolymer [35]. Thus, in the present study, the lignosulfonate superplasticizer does not efficiently play its role both on workability and compressive strength. The small effect on the workability (Figure 8) seems to be caused by the increase in water brought by the superplasticizer, not by any chemical effect of the additive. Therefore, it is suggested that lignosulfonate superplasticizer, which was developed for mixtures based on Portland cement, does not play its role when the binder is geopolymer due to a different reaction process under the high alkaline environment. Indeed, it was observed in a previous study that the superplasticizer loses its chemical properties in high alkaline media [36]. Thus, it is not interesting to use lignosulfonate superplasticizer for GRAC.

### 3.3. Microscopic Analyses

**Figure 11:**
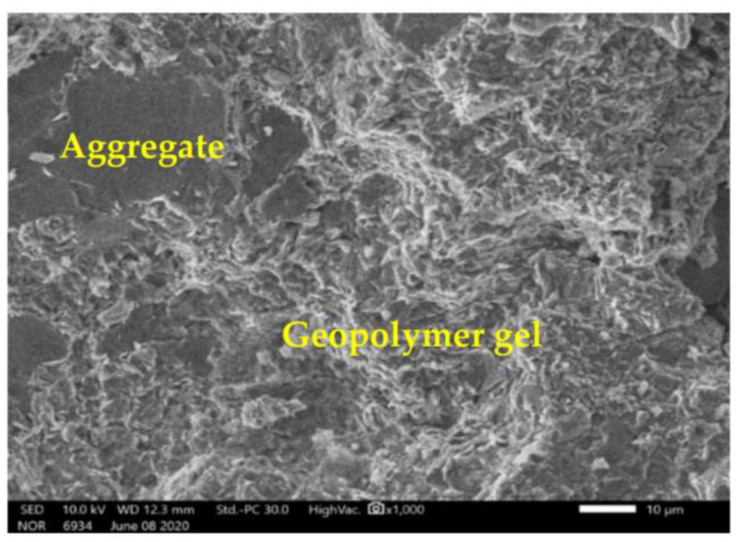
Development of geopolymer gels in GRAC.

The results from SEM show that the geopolymer gels have developed in the concrete specimens tested (Figure 11). The microstructure is relatively dense; no particular defect was noted.

**Figure 12:**
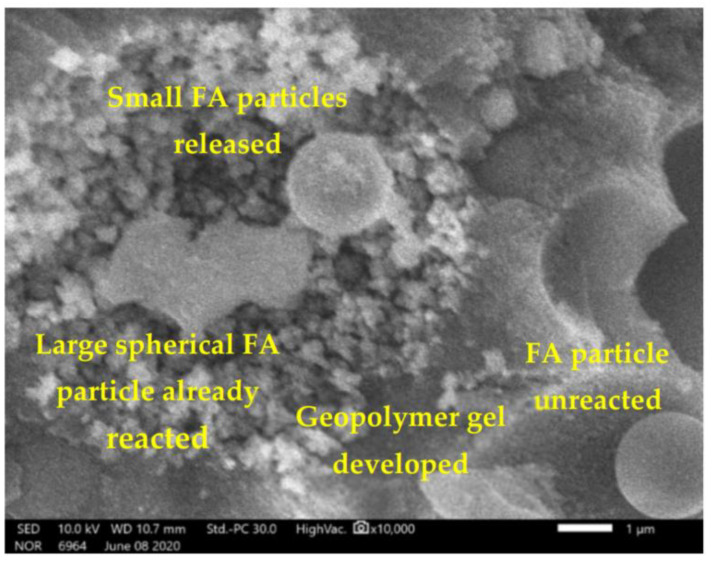
A large size FA particle already reacted; smaller FA particles still unreacted.

**Figure 13:**
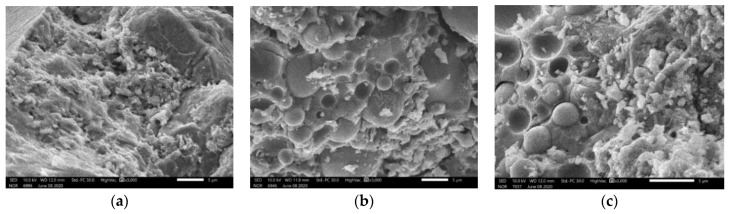
(**a**) FA completely reacted; (**b**) FA partially reacted (curing at ambient temperature); (**c**) FA partially reacted (curing with heating).

The large-size FA particles have participated in the reaction with AAS to create the geopolymer gels; however, there were still several small-size FA particles (less than 5 µm) which have not yet completely reacted (Figure 12) for both cases with or without heating treatment (Figure 13). Indeed, inside the big spherical FA particles there are small spherical FA particles [12]; thus, first the large spherical FA particles (covering) participated in the reaction with AAS, then the small FA particles (inside) were released. Figure 12 clearly shows this phenomenon (for the case of a specimen cured at ambient conditions). A portion of these small FA particles continued to participate to the reaction with AAS; other small FA particles were still unreacted. Nevertheless, for the case of specimens with heating treatment, there are less unreacted FA particles than the case of non-heating treatment (Figure 13). The unreacted FA particles (with small size, about 1 µm) can play the role as fillers.

**Figure 14:**
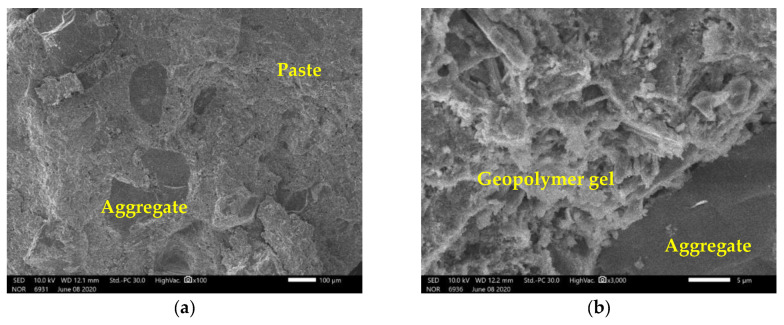
(**a**) Correct bonding between paste and aggregate, interfacial transition zone (ITZ) is not clear; (**b**) at a zoom: ITZ is not clear, geopolymer gel with tabular structure observed.

For cement concretes (both with natural aggregates or recycled aggregates), the ITZ between the cement paste and the aggregate is well-known as the zone of weakness. However, for specimens tested in the present study, the ITZ is not clear, but it appears that the aggregate is densely surrounded by the paste (Figure 14a). It is also observed that the bonding between the geopolymer paste and the aggregates is good (Figure 14b). From this figure, the geopolymer gel with tabular structure is also observed which is similar to that noted in the literature for high NaOH concentration (from 10 M) and at ambient temperature (27 °C) [37].

**Figure 15:**
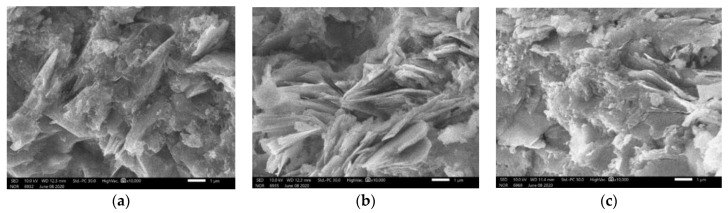
(**a–c**) Zoom of the geopolymer gels (at 1 µm) for specimens cured under ambient temperature.

**Figure 16:**
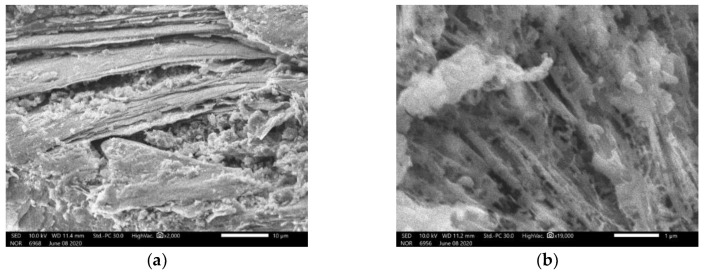
(**a**) Geopolymer gel with fiber form (specimen cured at 60 °C); (**b**) a zoom at 1 µm.

The zooms on the geopolymer gels (Figure 15) show that different form of links can be observed in the geopolymer gels for specimens cured under ambient temperature. For specimens cured at 60 °C, the geopolymer gels were created with fiber forms (Figure 16a). This form of gel is also similar to that observed in a previous study [37]. The zoom of the geopolymer gel presented in Figure 16b shows more clearly the fiber structure of the gel.

### 3.4. Compressive Strength of Geopolymer Mortar/Paste

**Figure 17:**
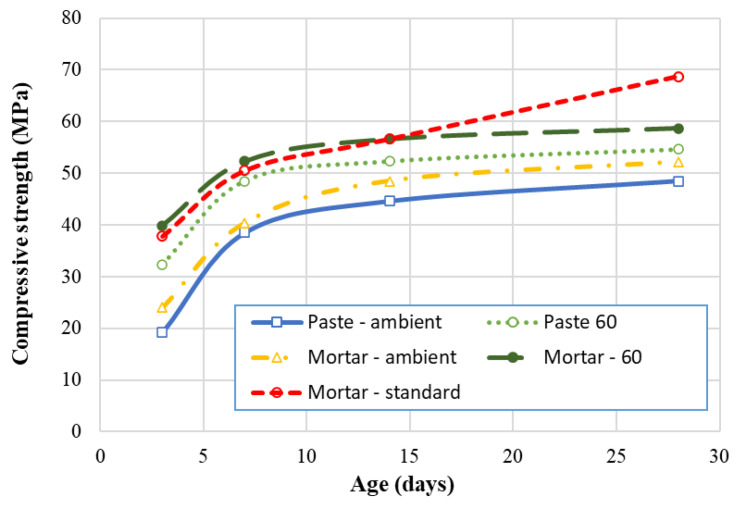
Compressive strength of geopolymer pastes/mortars.

In order to provide data for predicting the compressive strength of GRAC, experiments were conducted to determine the strength of geopolymer paste with an AAS/FA ratio = 0.4. The compressive strengths at 3, 7, 14, and 28 days are shown in Figure 17. The result shows a general evolution of the compressive strength of the mortar and paste specimens in function of the time; however, the evolution is different to that observed for GRAC. Indeed, a fast increase in compressive strength is observed until 7 days for geopolymer paste/mortar specimens, and then the evolution is slower. Then, a significant influence of the heating treatment on the compressive strength is observed: for both mortar specimens (with sand) and paste specimens (without sand), the heating treatment increases the compressive strength about 10–12% (par example by comparison between “Mortar–ambient”, and “Mortar–60” which means 60 °C of treatment). It is also worth noting that the addition of sand (case of mortar specimens) increased the compressive strength (compared to paste specimens).

At 28 days, the highest compressive strength was the “Mortar–standard” which had the mix proportion following the standardized cement mortar and containing the highest sand amount. These results suggest that the presence of sand improves the “skeleton” of the specimens, and therefore increased the compressive strength.

## 4. Model of Strength Prediction for GRAC

### 4.1. Classical Models for Portland Cement Concretes

Numerous classical models exist in the literature which propose the empirical formulas to predict the compressive strength of ordinary cement concrete. Among these models, Feret’s model is one of the most well-known thanks to its robustness [38]. Indeed, in this model, ordinary concrete is described as a two-phase material: a stiff inorganic inclusion (the aggregate) dispersed in a matrix (cement paste), considered homogeneous at the mesoscale. The compressive strength of the concrete at age *t*, called *f_c_*(*t*), is the function of the quality of the aggregates, represented by the empirical coefficient *K_g_*; the cement compressive strength at age *t*, called *f_cm_*(*t*); the water content *W*, the cement amount *C* (in mass), and the air volume *V_a_* existing in the final concrete obtained.

(2) $$f_{c}\left(t\right)=K_{g}f_{cm}\left(t\right)\frac{1}{{\left[1+\frac{\rho_{c}}{\rho_{w}}\left(\frac{W+\rho_{w}V_{a}}{C}\right)\right]}^{2}}$$

where:

*K_g_* is the Feret’s aggregate constant, 4.5 < *K*_g_ < 5.5 for natural aggregate.
*f_cm_*(*t*) is the compressive strength of cement at time *t*.
$\rho_{c}$ and $\rho_{w}$ are the specific densities of cement and water, respectively.
*W* and *C* is the mass of water and cement in 1 m^3^ of concrete.
*V_a_* is the volume of air for 1 m^3^ of concrete.

From the classical Feret’s model for ordinary cement concrete, De Larrard (1999) [39] has proposed a modified model which is more appropriate for new concretes such as high strength cement concrete or recycled aggregate cement concretes [40]. Indeed, the classical Feret’s model could not sufficiently describe the ceiling effect of some aggregates, especially in the case of high strength concrete where the compressive strength of concrete is not strictly proportional to the strength of the matrix. De Larrard’s model is an empirical hyperbolic equation taking into account this nonlinearity.

(3) $$f_{c}\left(t\right)=\frac{pf_{cm}\left(t\right)}{\left(qf_{cm28}+1\right)}$$

where:

*f_c_(t)* is the compressive strength of concrete at age *t*.
*f_cm_(t)* is the compressive strength of cement at age *t* (MPa).
*p* and *q* are the empirical coefficients which depend to the aggregate quality.

The objective of this study is to seek the values of *p* and *q* which are appropriate for the case of GRAC.

### 4.2. Compressive Strength of Geopolymer Paste

To apply the above Equations (2) and (3), the compressive strength of geopolymer paste *f_cm_(t)* must be determined. The results of geopolymer pastes/mortars with AAS/FA = 0.4 (at 28 days) are presented in Figure 17. In the above equations, *f_cm_(t)* is the compressive strength of the binder matrix (cement for ordinary cement concrete), therefore, for geopolymer concrete, it is logical that *f_cm_*(*t*) is the compressive strength of geopolymer paste (without sand). The results of geopolymer paste specimens are presented in Table 5.

### 4.3. Assessing the Relevancy of Feret’s Model

For geopolymer concrete where cement is not present and replaced by geopolymer binder (NaOH + Na_2_SiO_3_ + FA), the classical Feret’s model is proposed to be modified by replacing *C* (cement) in Equation (2) by *B* (binder). The modified Feret’s model is described as following:

(4) $$f_{c}\left(t\right)=K_{g}f_{cm}\left(t\right)\frac{1}{{\left[1+\frac{\rho_{b}}{\rho_{w}}\left(\frac{W+\rho_{w}V_{a}}{B}\right)\right]}^{2}}$$

The following parameters were used for the application of Feret’s model:

- The air volume *V_a_* of current non-air-entrained concrete is about 1–3% [41]; for geopolymer concrete, a value of 3.29% was indicated in a previous study [42]; so for the present study, *V_a_* of 3% was adopted, which corresponded to 30 liters of air in 1 m^3^ of concrete.
- The specific density of water $\rho_{w}$ is 1 t/m^3^. For the specific density of geopolymer binder $\rho_{b}$, as this value was not directly measured in the present study, a value of 2.6 t/m^3^ was adopted. Indeed, this value was estimated by taking into account the specific densities of FA, Na_2_SiO_3_, Na_2_O, and the compressive strength of the geopolymer paste obtained (from 50–70 MPa); this value is comparable to the results presented in a previous study [43].
- The mass of water *W* was determined from the waters existing in NaOH and Na_2_SiO_3_ solution, corresponding to 107.5 kg of water in 1 m^3^ of concrete;
- The mass of geopolymer binder *B*: this parameter replaces the cement amount *C* in the classical Feret’s model for ordinary cement concrete. Therefore, the mass of binder *B* should be the sum of mass of the solid parts (called *G*) in geopolymer binder in 1 m^3^ of concrete; this amount *G* corresponds to 50.8 kg Na_2_SiO_3_, 13.72 kg NaOH, and 428 kg FA, for a total of 492.5 kg. However, for geopolymer concrete, there may be differences with the ordinary cement concrete, so a coefficient *k_b_* was introduced to take into account the potential differences, so *B* is replaced by *k_b_G*. Indeed, as shown in the previous sections, there were still numerous FA particles which did not participate in the geopolymerization reactions, so the value of *k_b_* can vary from 0 to 1, which will be investigated in the present study.
- The Feret’s aggregate constant *K_g_*: the classical Feret’s model for natural aggregates indicated that *K_g_* could vary from 4.5 to 5.5 (high value of *K_g_* corresponds to good quality of aggregate). Some recent studies proposed some empirical relationships to calculate *K*_g_ from MDE (micro-Deval abrasion test) [44], which can be deduced from the aggregate crushing value (Section 2.1.3); however, the relevancy of these empirical formulas was also shown as limited [42]. For the present study, if that empirical relationship was applied, the value of *K_g_* obtained would be 7.7 and 6.4 for natural and recycled aggregates in the present study, respectively. These values are not logical when compared to the usual values mentioned above. For recycled aggregates with a lower quality than natural parent aggregates, the value of *K_g_* should be less than 4.5. The value of *K_g_* in the case of GRAC will be investigated in this study.
- For the ordinary cement concrete, *f_cm_*(*t*) is the compressive strength of cement at time *t*, so for GRAC the compressive strength of geopolymer paste (without sand) is used (Section 4.2).

**Figure 18:**
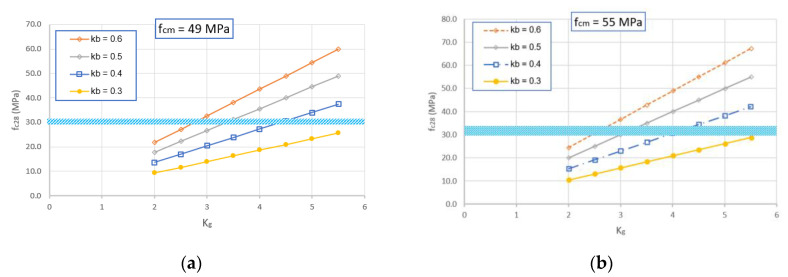
Numerical results of GRAC compressive strength at 28 days in function of *K_g_* and *k_b_*, for the case of ambient curing (**a**) and curing with heating treatment (**b**). The thick horizontal lines represent the experimental values.

The results obtained from the modified Feret’s model for compressive strength of GRAC at 28 days are illustrated in Figure 18. For the case of ambient curing, the experimental compressive strength of GRAC was of 29–31 MPa; from Figure 18a, the value identified of *K_g_* should be in an interval ranging from 2.8 to 4.3 with the corresponding values of *k_b_* from 0.4 to 0.6. For the case of curing at 60 °C (Figure 18b), the experimental compressive strength of geopolymer concrete was of 30–35 MPa; from this figure, the value identified of *K_g_* should be in the interval ranging from 2.5 to 4.3 with the corresponding values of *k_b_* from 0.4 to 0.6. By taking into account the quality of the recycled aggregates used (lower quality than natural aggregates), the values of *K_g_* identified (from 2.5 to 4.3, which are lower than the current cases of natural aggregates) seem to be logical.

**Figure 19:**
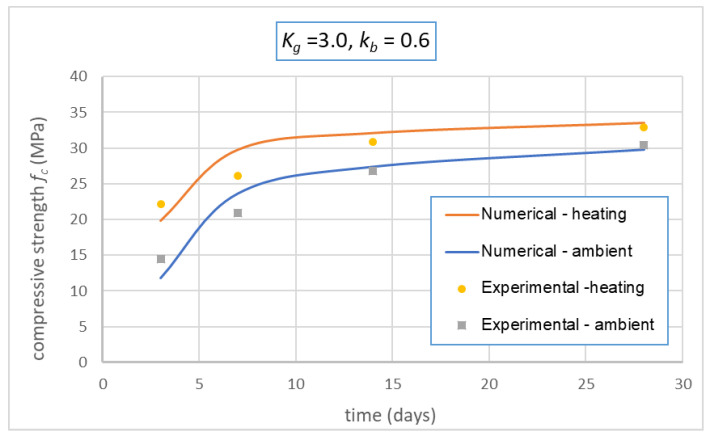
Comparison of experimental and numerical results obtained by Feret’s model for GRAC at different ages.

**Figure 20:**
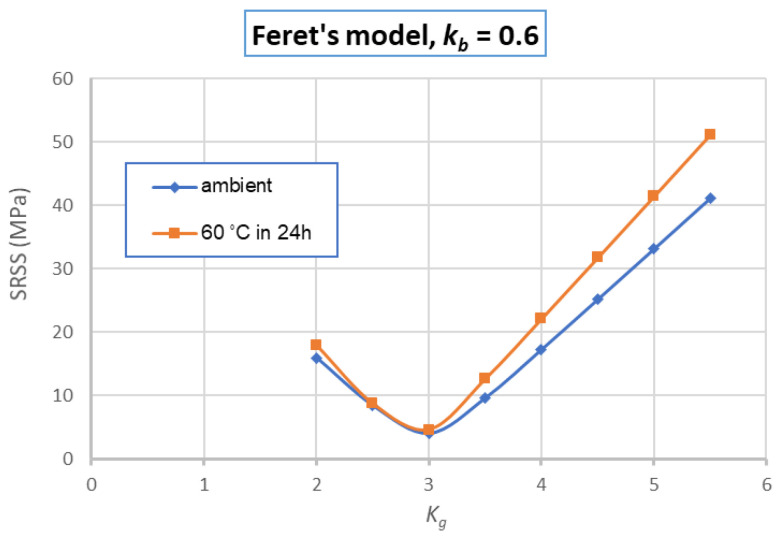
Variation of square root of the sum of the squares (SRSS) of GRAC compressive strength (for 3, 7, 14, and 28 days) in function of *K_g_* (for *k_b_* = 0.6).

To verify the relevancy of the modified Feret’s model and to identify best values of coefficients *K_g_* and *k_b_*, the model was applied for the compressive strength of GRAC at other days (3, 7, and 14). Figure 19 presents an example of the numerical results obtained for the case *K_g_ =* 3.0, *k_b_ =* 0.6. Then the SRSS (square root of the sum of the squares) of the differences between the numerical and experimental results was calculated for each case of *K_g_* and *k_b_*; the values of *K_g_* and *k_b_* which provide the lowest values of SRSS were chosen. Figure 20 illustrates example of the variation of SRSS in function of *K_g_* and *k_b_* for the case of *k_b_ =* 0.6. From this figure, the best values should be *K_g_ =* 3.0, because they provide the lowest values of SRSS for both cases of curing in ambient conditions and with heating treatment. Thus, the values identified are *K_g_ =* 3.0, *k_b_ =* 0.6. The value identified of the binder coefficient *k_b_* (*=* 0.6) can be explained by two reasons: first, the components in FA which participated to the polymerization reactions are *Al_2_O_3_* and *SiO_2_* [45] which are 77% of FA (Table 1); second, there were still several FA particles and AAS which could not participate to the reactions due to the homogeneity of the materials during the mixing. The numerical results obtained with these coefficients are compared with the experimental results and presented in Figure 19. This figure shows that the modified Feret’s model with the coefficients identified can reproduce the compressive strength of GRAC at 14 and 28 days with high accuracy (differences less than 1 MPa); the differences are more significant for 7 and 14 days but still less than 3 MPa.

**Figure 21:**
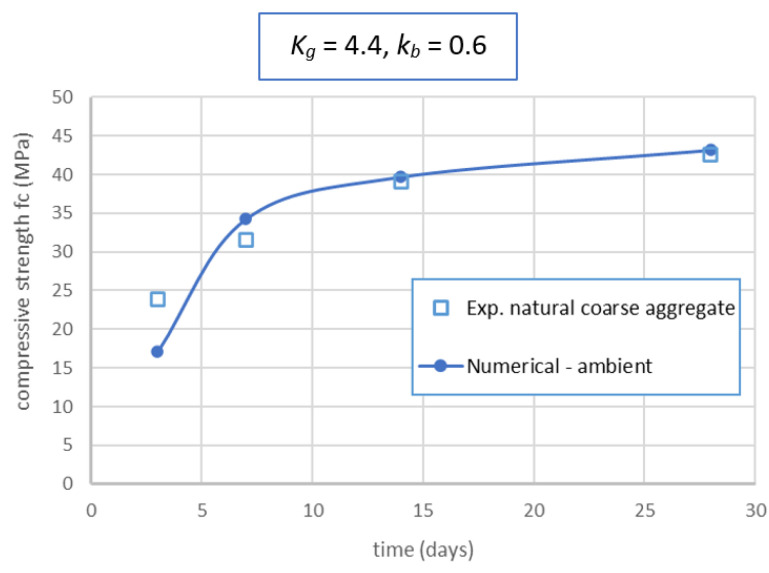
Comparison of experimental and numerical results obtained by modified Feret’s model for geopolymer concrete with natural coarse aggregate.

To verify again the robustness of the modified Feret’s model and the parameters identified, the specimens of geopolymer concrete with natural coarse aggregates were manufactured and tested. These specimens had the same proportions and properties as GRAC; only the natural coarse aggregate was used in place of recycled concrete aggregates. These specimens were cured at ambient conditions. In application of the modified Feret’s model for this geopolymer concrete, the binder coefficient *k_b_* was taken as the same as the value identified previously (*=* 0.6); only the aggregate coefficient *K_g_* varied. The best value of *K_g_* was also identified by the SRSS approach, from which a value of *K_g_* = 4.4 was identified. The comparison between the experimental and numerical results are presented in Figure 21. This figure shows the relevancy of the modified Feret’s model for the case of geopolymer concrete. The value identified (*K_g_* = 4.4) is also logical when compared to the current values of *K_g_* for ordinary concretes with natural aggregates. Indeed, as mentioned in the classical Feret’s model, the current values of *K_g_* for natural aggregates are from 4.5 to 5.5.

### 4.4. Relevancy of Modified De Larrard’s Model

**Figure 22:**
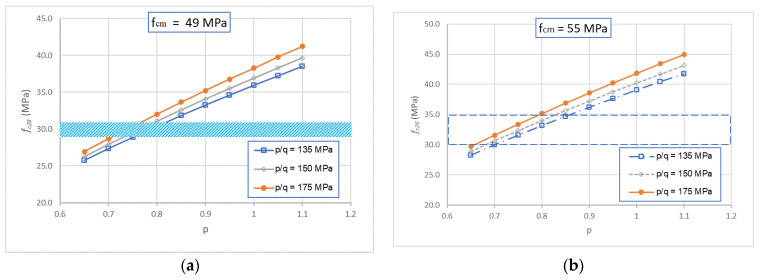
Numerical results of concrete compressive strength in function of p and p/q, for the case of ambient curing (**a**) and curing with heating treatment (**b**). The thick horizontal lines represent the experimental values.

To apply De Larrard’s model, the parameters *p* and *q* must be determined. In a previous study, the aggregate parameters *p* and *q* were identified for natural and recycled aggregates [39]. It was observed that depending to the quality of each aggregate, the values of *p* could vary about from 0.65 to 1.2, and *p*/*q* could vary about from 120 to 200, respectively. In the present study, to identify the values of *p* and *p*/*q* corresponding to the recycled aggregate used, a parametric study was performed: *p* varied from 0.65 to 1.1, and at the same time *p*/*q* varied from 135 to 175. The compressive strength at 28 days of geopolymer recycled aggregate concretes was determined by applying Equation (3) and by using the corresponding compressive strength of geopolymer pastes (*f_cm_*_28_, for the cases of ambient curing or with heating treatment). The results are illustrated in Figure 22.

For the case of ambient curing, the experimental compressive strength of geopolymer concrete was of 29–31 MPa. From Figure 22a, the value identified of *p* should be about 0.75; the change in *p*/*q* does not influence significantly the compressive strength obtained. We proposed to take *p*/*q* = 175; this value will be checked for other cases.

**Figure 23:**
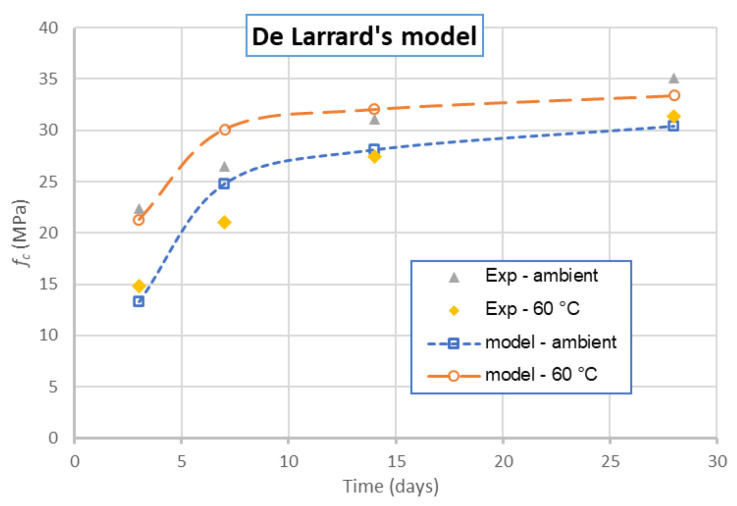
Comparison of numerical and experimental results of GRAC compressive strength at different ages.

For the case of curing at 60 °C, the experimental compressive strength of geopolymer concrete was of 30–35 MPa. From Figure 22b, the value identified of *p* should be around 0.75; the change of *p*/*q* does not vary significantly from the compressive strength obtained. Thus, the previous proposition of *p* = 0.75 and *p*/*q* = 175 is also acceptable for this case. This result is interesting because the values of *p* = 0.75 and *p*/*q* = 175 can reproduce the compressive strength for different cases: with or without thermal treatment during the curing, with or without using the plasticizer. It is logical that *p* and *p*/*q* depend only on the aggregate properties. Thus, the difference in the compressive strength obtained for GRAC is the compressive strength of the geopolymer paste. These values are also checked for other cases which means the compressive strength of GRAC at other ages. The results are presented in Figure 23.

This figure shows that De Larrard’s model with the parameters *p* and *q* identified in the present study can be reproduced with acceptable accuracy for the compressive strength of GRAC at 3, 14, and 28 days (differences of about 1 MPa between the experimental results and the numerical ones). For the case of 7 days, the differences are more remarkable. Indeed, the model predicts a higher increase in compressive strength from 3 to 7 days (similar to the case of geopolymer paste and mortar) but the experimental results show that the strength evolution from 3 to 7 days is slower. For practice application, when compared to the modified Feret’s model, De Larrard’s model is easier because it demands fewer parameters.

## 5. Conclusions and Prospects

The recycling in general and the recycled aggregate concrete are important topics which attract attention in the context of sustainable development and circular economy. The use of an alternative binder for recycled aggregate concrete is also a subject which needs to be investigated. In the present study, the geopolymer recycled aggregate concrete was studied; the effect of different AAS/FA ratios, curing temperature, LignoSulfonate superplasticizer on the workability, and the compressive strengths of GRAC were evaluated. Two empirical models were also proposed and assessed.

The results showed encouraging results on the use of recycled coarse aggregate (at 100% replacement) and the fly ash-based alkali-activated binder for the manufacture of concrete. The increase in AAS/FA ratio from 0.4 to 0.5 increased the slump and decreased the compressive strength of the concrete obtained; the mean change observed was about a 10% increase in slump and 10% decrease in compressive strength. It is worth noting that an economic calculation shows that the price of GRAC (with the mix design used in the present study) is really equivalent to that of an ordinary cement concrete in Vietnam (about 48 USD per m^3^). This result shows the possible application in short term of GRAC.

It was observed in this study that the addition of lignosulfonate superplasticizer had a low effect on the slump and no significant effect on the compressive strength of GRAC. The concrete achieves higher strength compressive when cured at 60 °C instead of curing at ambient temperature. The analyses at microscopic scale showed that the large-size FA particles participated in the reactions to create geopolymer gels, while numerous smaller FA particles (about 1 µm) did not completely react. The results also showed that there was correct bonding between the geopolymer gels and the aggregates, which is a positive point when compared to the ITZ in Portland cement concretes.

Finally, the models for the prediction of compressive strength were investigated which were based on Feret’s and De Larrard’s models. For Feret’s model, several adaptations were necessary to adopt a model which was developed for ordinary cement concrete and now applied for a geopolymer concrete. For De Larrard’s model, the parameters *p* and *q* have been identified for the recycled aggregates used. The results showed that both models used could be reproduced with satisfying accuracy for most of experimental results. Therefore, these models can provide useful information for the mix design and compressive strength prediction of GRAC. Further studies on the application of these models for GRAC will be interesting.

## Figures and Tables

**Table 1:** Chemical composition of FA used.

Components	% in Mass
Sulfur trioxode (SO_3_)	1.0
Aluminum oxide (Al_2_O_3_)	26.1
Ferric oxide (Fe_2_O_3_)	11.3
Sodium oxide (Na_2_O)	1.35
Silicon dioxide (SiO_2_)	51.1
Potassium oxide (K_2_O)	1.29
Calcium oxide (CaO)	4.7
Magnesium oxide (MgO)	1.7
Moisture	0.1
Loss on ignition	0.7

**Table 2:** Characteristics of aggregates studied.

	Specific Gravity	Dry Density	Saturated Density	Water Absorption	ACV (Saturated)	Compressive Strength (MPa)
Natural aggregates	2.66	2.59	2.61	1.1%	15.0%	70
Recycled coarse aggregates	2.60	2.26	2.39	5.8%	25.8%	34

**Table 3:** Mix proportions of geopolymer recycled aggregate concrete (kg/m^3^).

AAS/FA	FA	Na_2_SiO_3_	NaOH	Sand	RCA	Superplasticizer
0.4	428	123	49	540	1260	8.28
0.45	414	133	53	540	1260	8.00
0.5	400	143	57	540	1260	7.74

**Table 4:** Mix summary the proportion of the geopolymer mortar/paste.

Composition (%)	Paste	Mortar	Mortar-Standard
FA	71.3	37.5	22.7
AAS	28.7	15.1	9.1
Sand	0	47.4	68.2

**Table 5:** Compression strength of geopolymer paste (MPa).

Cured Conditions	Age (Days)			
	3	7	14	28
Ambient	19.2	38.5	44.6	48.5
60 °C in 24 h	32.3	48.5	52.3	54.6

## Data Availability

The data presented in this study are available on request from the corresponding author.
